# High-Sensitivity Cardiac Troponin and the Universal Definition of Myocardial Infarction

**DOI:** 10.1161/CIRCULATIONAHA.119.042960

**Published:** 2019-10-07

**Authors:** Andrew R. Chapman, Philip D. Adamson, Anoop S.V. Shah, Atul Anand, Fiona E. Strachan, Amy V. Ferry, Kuan Ken Lee, Colin Berry, Iain Findlay, Anne Cruikshank, Alan Reid, Alasdair Gray, Paul O. Collinson, Fred Apple, David A. McAllister, Donogh Maguire, Keith A.A. Fox, Catalina A. Vallejos, Catriona Keerie, Christopher J. Weir, David E. Newby, Nicholas L. Mills

**Affiliations:** 1 BHF Centre for Cardiovascular Science (A.R.C., P.D.A., A.S.V.S., A.A., F.E.S., A.V.F., K.K.L., K.A.A.F., D.E.N., N.L.M.), University of Edinburgh, United Kingdom.; 2 Edinburgh Clinical Trials Unit (C.K., C.J.W.), University of Edinburgh, United Kingdom.; 3 MRC Human Genetics Unit (C.A.V.), University of Edinburgh, United Kingdom.; 4 Usher Institute of Population Health Sciences and Informatics (C.K., C.J.W., N.L.M.), University of Edinburgh, United Kingdom.; 5 Christchurch Heart Institute, University of Otago, New Zealand (P.D.A.).; 6 Institute of Cardiovascular and Medical Sciences (C.B.), University of Glasgow, United Kingdom.; 7 Institute of Health and Wellbeing (D.A.McA.), University of Glasgow, United Kingdom.; 8 Department of Cardiology, Royal Alexandra Hospital, Paisley, United Kingdom (I.F.).; 9 Department of Biochemistry, Queen Elizabeth University Hospital, Glasgow, United Kingdom (A.C., A.R.).; 10 Emergency Medicine Research Group Edinburgh, Royal Infirmary of Edinburgh, United Kingdom (A.G.).; 11 Departments of Clinical Blood Sciences and Cardiology, St George’s, University Hospitals NHS Trust and St George’s University of London, United Kingdom (P.O.C.).; 12 Department of Laboratory Medicine and Pathology, Hennepin Healthcare/Hennepin County Medical Center & University of Minnesota, Minneapolis (F.A.).; 13 Emergency Medicine Department, Glasgow Royal Infirmary, United Kingdom (D.M).; 14 The Alan Turing Institute, London, United Kingdom (C.A.V.).

**Keywords:** myocardial infarction, troponin

## Abstract

**Background:**

The introduction of more sensitive cardiac troponin assays has led to increased recognition of myocardial injury in acute illnesses other than acute coronary syndrome. The Universal Definition of Myocardial Infarction recommends high-sensitivity cardiac troponin testing and classification of patients with myocardial injury based on pathogenesis, but the clinical implications of implementing this guideline are not well understood.

**Methods:**

In a stepped-wedge cluster randomized, controlled trial, we implemented a high-sensitivity cardiac troponin assay and the recommendations of the Universal Definition in 48 282 consecutive patients with suspected acute coronary syndrome. In a prespecified secondary analysis, we compared the primary outcome of myocardial infarction or cardiovascular death and secondary outcome of noncardiovascular death at 1 year across diagnostic categories.

**Results:**

Implementation increased the diagnosis of type 1 myocardial infarction by 11% (510/4471), type 2 myocardial infarction by 22% (205/916), and acute and chronic myocardial injury by 36% (443/1233) and 43% (389/898), respectively. Compared with those without myocardial injury, the rate of the primary outcome was highest in those with type 1 myocardial infarction (cause-specific hazard ratio [HR] 5.64 [95% CI, 5.12–6.22]), but was similar across diagnostic categories, whereas noncardiovascular deaths were highest in those with acute myocardial injury (cause specific HR 2.65 [95% CI, 2.33–3.01]). Despite modest increases in antiplatelet therapy and coronary revascularization after implementation in patients with type 1 myocardial infarction, the primary outcome was unchanged (cause specific HR 1.00 [95% CI, 0.82–1.21]). Increased recognition of type 2 myocardial infarction and myocardial injury did not lead to changes in investigation, treatment or outcomes.

**Conclusions:**

Implementation of high-sensitivity cardiac troponin assays and the recommendations of the Universal Definition of Myocardial Infarction identified patients at high-risk of cardiovascular and noncardiovascular events but was not associated with consistent increases in treatment or improved outcomes. Trials of secondary prevention are urgently required to determine whether this risk is modifiable in patients without type 1 myocardial infarction.

**Clinical Trial Registration:**

https://www.clinicaltrials.gov. Unique identifier: NCT01852123.

## Abstract 2

Supplemental Digital Content is available in the text.

**Clinical Perspective**

**What Is New?**

- No previous randomized, controlled trials have evaluated the effect of implementing a high-sensitivity cardiac troponin assay and the recommendations of the Universal Definition of Myocardial Infarction on the investigation, treatment, and outcomes of patients stratified according to the proposed diagnostic classification.
- We demonstrate that implementation of high-sensitivity cardiac troponin testing leads to a disproportionate increase in type 2 myocardial infarction and myocardial injury.
- We found all patients with myocardial injury and infarction are at increased future cardiovascular risk, irrespective of pathogenesis, despite an excess in noncardiovascular death in patients with type 2 myocardial infarction and myocardial injury.

**What Are the Clinical Implications?**

- Clinicians should consider investigations to define coronary or structural heart disease in patients with type 2 myocardial infarction and myocardial injury.
- The risk of future cardiovascular events should be evaluated on an individual patient basis using all available clinical information.
- Until randomized, controlled trials are available, secondary prevention therapies should be considered on a pragmatic basis with the aim of reducing future cardiovascular risk.

**Editorial, see p 172**

The Universal Definition of Myocardial Infarction has evolved to accommodate improvements in the sensitivity of cardiac troponin assays.^1–3^ This international guideline recommends the use of high-sensitivity cardiac troponin (hs-cTn) assays and the 99^th^ centile upper reference limit as the diagnostic threshold for myocardial infarction.^4–7^ It also recognizes that myocardial injury occurs in many conditions other than acute coronary syndromes,^8–11^ and therefore proposes additional criteria for the classification of patients with myocardial injury and infarction based on pathogenesis.^2,12^

Despite nearly half of all elevations in cardiac troponin occurring in patients with type 2 myocardial infarction or myocardial injury,^13^ this classification and its consequences for patients are not well understood in practice. We recently reported the primary outcome from a multicenter randomized, controlled trial evaluating the impact of implementing a hs-cTnI assay on clinical outcomes in consecutive patients with suspected acute coronary syndrome.^14^ The introduction of hs-cTn reclassified 1 in 6 patients with myocardial necrosis who were not identified by the previous generation troponin assay, but this was not associated with an improvement in outcomes. In this prespecified secondary analysis of the trial, we report whether implementing hs-cTn testing and the recommendations of the Universal Definition of Myocardial Infarction led to changes in investigation, treatment and outcomes in patients stratified according to the proposed diagnostic classification.

## Methods

### Transparency and Openness Promotion

The trial makes use of multiple routine electronic health care data sources that are linked, deidentified, and held in our national safe haven, which is accessible by approved individuals who have undertaken the necessary governance training. Summary data and the analysis code can be made available upon request from the corresponding author.

### Study Population and Trial Design

High-STEACS (High-Sensitivity Troponin in the Evaluation of Patients With Suspected Acute Coronary Syndrome) is a stepped-wedge cluster randomized, controlled trial to evaluate implementation of a hs-cTn I assay and the recommendations of the Universal Definition of Myocardial Infarction in consecutive patients with suspected acute coronary syndrome, across 10 secondary and tertiary care hospitals in Scotland. All patients attending the emergency department were screened for suspected acute coronary syndrome by the attending clinician at the time troponin was requested, using an electronic form integrated into the clinical care pathway. Patients were eligible for inclusion if they presented with suspected acute coronary syndrome and had paired cardiac troponin measurements from the standard care and trial assay. Patients were excluded if they had been admitted previously during the trial period or were not resident in Scotland.

### Randomization

All sites reported cardiac troponin using a contemporary troponin assay and existing diagnostic threshold in a validation phase of at least 6 months, before being randomly allocated to early or late implementation of a high-sensitivity assay with sex-specific 99^th^ centile thresholds as recommended by the Universal Definition (Appendix A in the online-only Data Supplement).

### Intervention

Cardiac troponin testing was performed at presentation and was repeated 6 or 12 hours after the onset of symptoms at the discretion of the attending physician and in accordance with national guidelines.^15^ In the validation phase, a contemporary cTnI assay (Abbott Laboratories, Abbott Park, IL, USA) was used to guide clinical decisions. The inter-assay coefficient of variation was determined at each site and was less than 10% at 40 ng/L (7 sites) and 50 ng/L (3 sites). Only cTnI concentrations above these diagnostic thresholds were reported. During the implementation phase, a hs-cTnI assay (ARCHITECT_*STAT*_ high-sensitive troponin I assay; Abbott Laboratories, Abbott Park, IL, USA) was used to guide clinical decisions. This assay has an inter-assay coefficient of variation of less than 10% at 4.7 ng/L, and a 99^th^ centile upper reference limit of 34 ng/L in men and 16 ng/L in women.^4^

To support implementation, we provided written educational material and presentations at each site and updated the electronic patient record to highlight the change in assay and diagnostic thresholds. Educational material on the new assay, decision thresholds, and on the classification of myocardial injury and infarction were presented at each emergency department handover (twice daily). A detailed summary of the trial procedures and intervention is available in Appendix A in the online-only Data Supplement.

### Adjudication of Myocardial Injury and Infarction

All diagnoses in patients with hs-cTnI concentrations above the 99^th^ centile were adjudicated and classified according to the Third Universal Definition of Myocardial Infarction.^1^ In this prespecified secondary analysis, this classification was updated to the Fourth Universal Definition of Myocardial Infarction.^2^ Two physicians independently reviewed all clinical information, blinded to study phase, with discordant diagnoses resolved by a third reviewer. Type 1 myocardial infarction was defined as myocardial necrosis (any hs-cTnI concentration above the sex-specific 99th centile with a rise or fall in hs-cTnI concentration where serial testing was performed) in the context of a presentation with suspected acute coronary syndrome with symptoms or signs of myocardial ischemia on the electrocardiogram. Patients with myocardial necrosis, symptoms or signs of myocardial ischemia, and evidence of increased myocardial oxygen demand or decreased supply secondary to an alternative condition without evidence of acute atherothrombosis were defined as type 2 myocardial infarction. Patients with hs-cTnI concentrations above the 99^th^ centile without symptoms or signs of myocardial ischemia were classified as having myocardial injury. The final clinical diagnosis was also adjudicated according to prespecified criteria. All non-ischemic myocardial injury was classified as acute, unless a change of ≤20% was observed on serial testing,^2^ or the final adjudicated diagnosis was chronic heart failure or chronic renal failure, where the classification was chronic myocardial injury. A detailed summary of the adjudication procedures is provided in Appendix B in the online-only Data Supplement.

### Trial Outcomes

We used regional and national registries to ensure complete follow-up for the trial population.^16^ The primary outcome was myocardial infarction (type 1 or type 4b) or cardiovascular death at 1 year. Primary outcome events were adjudicated by a panel who were blinded to the index presentation and study phase (Appendix A in the online-only Data Supplement). Secondary outcomes included all-cause death, cardiovascular death, cardiac death, noncardiovascular death, duration of stay, myocardial infarction (type 1 or type 4b), unplanned coronary revascularization, hospitalization for heart failure, ischemic stroke, major hemorrhage and unplanned hospitalization.

### Ethical Approval

The study was approved by the Scotland A Research Ethics Committee, the Public Benefit and Privacy Panel for Health and Social Care, and by each National Health Service Health Board. All data were collected prospectively from the electronic patient record, deidentified and linked within secure National Health Service Safe Havens.

### Patient and Public Involvement

Both patients and lay representatives were members of the trial steering committee for the High-STEACS clinical trial and all related studies (NCT: 01852123) and were involved in the design, conduct and approval of this study.

### Statistical Analysis

Baseline characteristics were summarized for the study population and in groups according to the universal definition classification. We assessed agreement between adjudicators across diagnostic categories using Cohen’s Kappa. Group-wise comparisons were performed using χ^2^, Kruskal–Wallis or 1-way analysis of variance tests as appropriate. In post hoc analysis, we compared management by classification, with type 1 myocardial infarction as the reference group, including a Bonferroni correction for multiple testing. Based on previous observations of an excess in noncardiovascular death in patients with type 2 myocardial infarction and myocardial injury,^11^ we applied competing risks methodology in all analyses.^17^ The risk of the primary outcome and competing risk of noncardiovascular death was estimated using a cumulative incidence function.^18^ Outcome rates were compared between groups using cause-specific hazard ratios (csHR) obtained from a Cox regression model, with patients without myocardial injury as the referent group. The model was adjusted for age, sex, a history of ischemic heart disease or diabetes mellitus, renal function (creatinine concentration), time of presentation from the start date of the trial, season, site of recruitment (as a random effect), and phase of the trial. Where data were missing for creatinine concentration (1.9%, 948/48 282) this was assumed to be at random, and multiple imputation was applied using chained equations with 5 imputations of the data set. We compared the rates of the primary outcome before and after implementation of the high-sensitivity assay by group using an identical Cox proportional hazards model, including additional terms for the log transformed peak troponin concentration and interaction terms for phase of the trial and diagnostic group. All analyses were prespecified (Appendix C in the online-only Data Supplement) and performed in R (Version 3.5.1) using the *survival* and *cmprsk* packages.

## Results

### Trial Sites and Population

We enrolled 48 282 patients (61±17 years, 47% women) with suspected acute coronary syndrome across 10 sites with 39% (18 978/48 282) and 61% (29 304/48 282) enrolled during the validation and implementation phase, respectively.

### Classification by the Universal Definition of Myocardial Infarction

**Figure 1.**
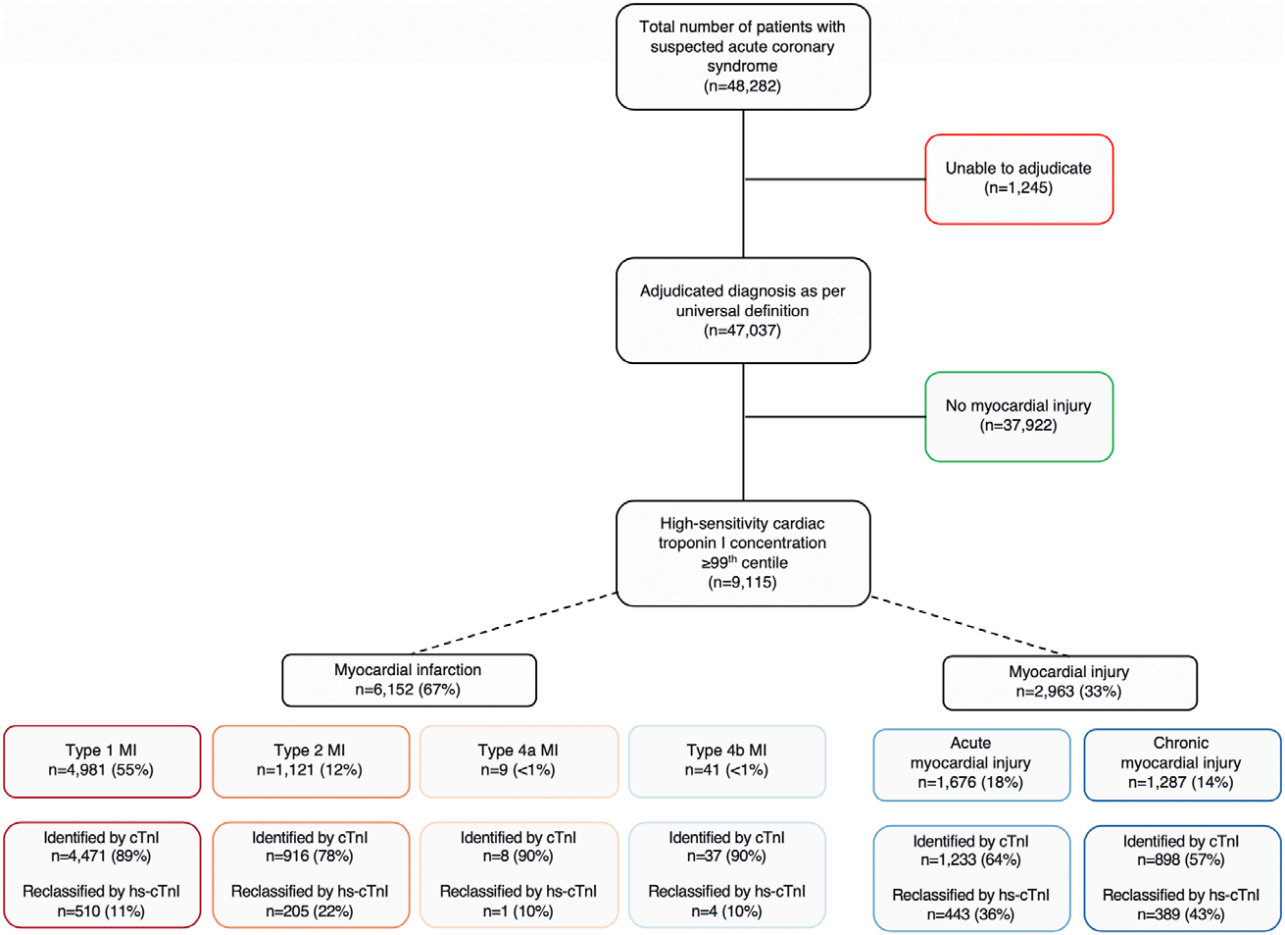
**Consort diagram with identification of the study population by classification, and proportion identified by the contemporary troponin assay (cTnI) or reclassified by the high-sensitivity assay (hs-cTnI).** Serial cardiac troponin concentrations were available in 77% (6983 of 9115) of patients with myocardial injury. MI indicates myocardial infarction.

During the index presentation, 21% (10 360/48 282) of patients had hs-cTnI concentrations above the 99^th^ centile (Figure 1). It was possible to adjudicate the diagnosis in 88% (9115/10 360) of patients (Table I in the online-only Data Supplement), and there was substantial agreement between adjudicators (K=0.75). The adjudicated diagnosis was type 1 myocardial infarction in 55% (4981/9115), type 2 myocardial infarction in 12% (1121/9115), and acute or chronic myocardial injury in 18% (1676/9115) and 14% (1287/9115), respectively (Table 1). Diagnostic agreement was substantial in patients with type 1 myocardial infarction (K=0.78) and myocardial injury (K=0.65) but was lower in those with type 2 myocardial infarction (K=0.49). Compared with the Third Universal Definition,^14^ adoption of the recommendations from the Fourth Universal Definition reclassified 15% (1320/9115) of patients, with the majority of those reclassified having chronic myocardial injury (Figure I in the online-only Data Supplement). The use of objective criteria for myocardial oxygen supply or demand imbalance as proposed in the Fourth Definition led to an improvement in diagnostic agreement in those with type 2 myocardial infarction (K=0.62).

**Table 1.**
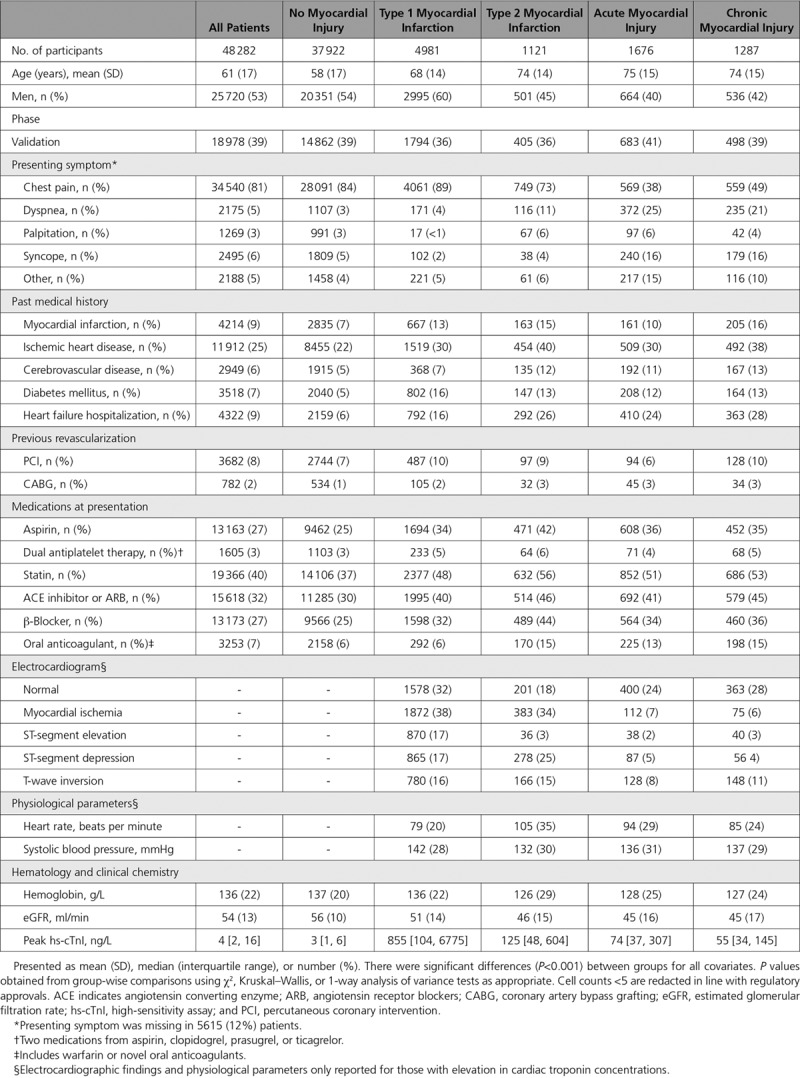
Characteristics of the Trial Participants Classified by the Fourth Universal Definition of Myocardial Infarction

Compared with those with type 1 myocardial infarction, patients with type 2 myocardial infarction were older (74±14 vs 68±14 years), were more likely to be women (55% vs 40%), and more likely to have a history of cardiovascular disease. Patients with acute or chronic myocardial injury were of similar age and sex to those with type 2 myocardial infarction. Peak troponin concentrations were higher in patients with type 1 myocardial infarction (855 ng/L, interquartile range (IQR) 104–6775 ng/L) compared with type 2 myocardial infarction (125 ng/L, IQR 48–604 ng/L) and either acute or chronic myocardial injury (74 ng/L, IQR 37–307 ng/L, and 55 ng/L, IQR 34–145 ng/L, respectively; Table 1).

### Management by Classification

The majority of patients with type 1 myocardial infarction were started on antiplatelet or anticoagulant therapy in the emergency department (55%) and had coronary angiography (59%) or percutaneous coronary intervention (41%) during the index presentation (Table 2). Patients were likely to receive additional treatments including single (67%) or dual (60%) antiplatelet, angiotensin-converting enzyme inhibitor or angiotensin receptor blocker (32%), beta-blocker (38%) or statin (35%) therapies. Fewer patients with type 2 myocardial infarction received antiplatelet or anticoagulant therapy at presentation (26%) or underwent coronary angiography (10%) and percutaneous coronary intervention (2%; *P*<0.001 for all). On discharge, patients with type 2 myocardial infarction were less likely to receive new treatment with single (19%) or dual (10%) antiplatelet, angiotensin-converting enzyme inhibitor or angiotensin receptor blocker (9%), beta-blocker (20%) or statin (6%) therapies (*P*<0.001 for all). Patients with acute or chronic myocardial injury received fewer therapies than patients with type 1 or type 2 myocardial infarction (*P*<0.001 for all).

**Table 2.**
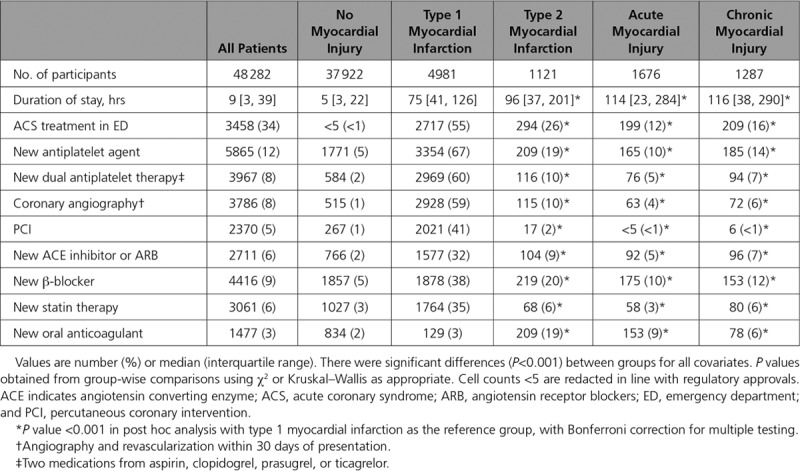
Management During Index Hospital Admission Stratified by the Fourth Universal Definition of Myocardial Infarction

### Outcomes by Classification

**Figure 2.**
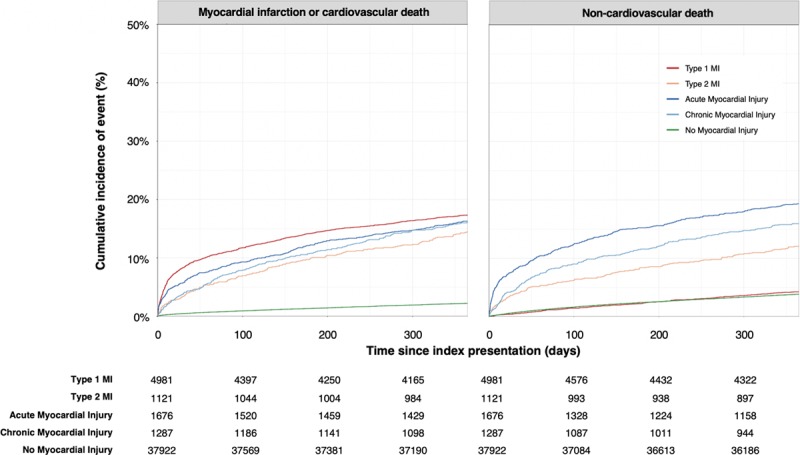
**Cumulative incidence curves for the primary outcome of type 1 or 4b myocardial infarction or cardiovascular death, and competing risk of noncardiovascular death, stratified by type 1 myocardial infarction (red), type 2 myocardial infarction (gold), acute myocardial injury (dark blue), chronic myocardial injury (light blue), and no myocardial injury (green) with table of number at risk.** Estimates obtained from a cumulative incidence function. MI indicates myocardial infarction.

**Figure 3.**
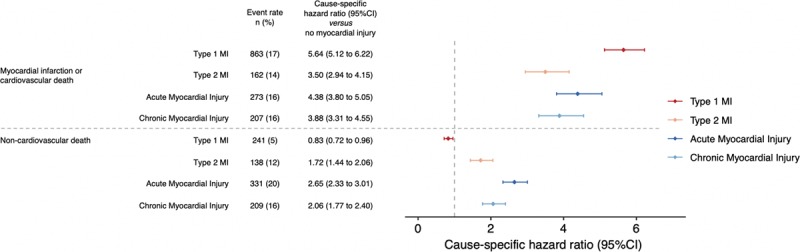
**Forest plot of the primary outcome (type 1 or 4b myocardial infarction or cardiovascular death) and noncardiovascular death in the trial population stratified by index diagnosis; type 1 myocardial infarction (red), type 2 myocardial infarction (gold), acute myocardial injury (dark blue) and chronic myocardial injury (light blue), relative to those with no myocardial injury.** Adjusted cause-specific hazard ratios (csHR) obtained from multivariable cox regression models including adjustment for age, sex, a history of ischemic heart disease or diabetes mellitus, renal function, time of presentation from the start date of the trial, season, phase of the trial, and site of recruitment (as a random effect). In this model the competing event or time of censor are both considered as independent outcomes. MI indicates myocardial infarction.

The primary outcome of subsequent myocardial infarction or cardiovascular death occurred in 17% (863/4981) of patients with type 1 myocardial infarction, 14% (162/1121) with type 2 myocardial infarction, 16% (273/1676) with acute myocardial injury, and 16% (207/1287) with chronic myocardial injury (Table II in the online-only Data Supplement). When compared with those without myocardial injury, the risk and rate of the primary outcome was highest in patients with type 1 myocardial infarction (Figure 2; csHR 5.64 [95% CI, 5.12–6.22]), but increases were also observed in patients with type 2 myocardial infarction (csHR 3.50 [95% CI, 2.94–4.15]), acute myocardial injury (csHR 4.38 [95% CI, 3.80–5.05]) and chronic myocardial injury (csHR 3.88 [95% CI, 3.31–4.55], Figure 3, Table III in the online-only Data Supplement). The rate of future type 1 or 4b myocardial infarction at 1 year was highest in those with an index type 1 myocardial infarction (9%, 466/4981), and lower in those with type 2 myocardial infarction (5%, 51/11121), and acute (3%, 56/1676) or chronic myocardial injury (4%, 57/1287).

**Figure 4.**
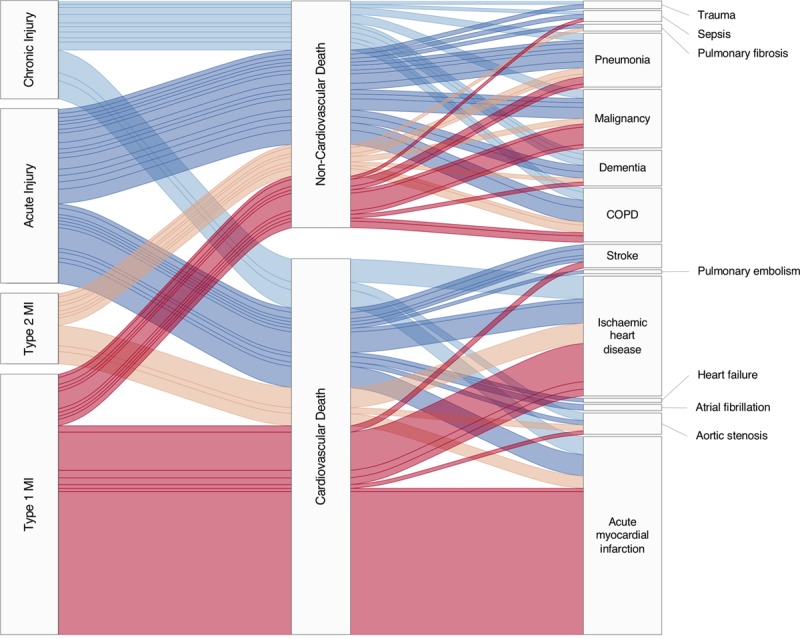
**Flow diagram (alluvial plot) illustrating the frequency of cause of death grouped by classification of myocardial infarction and cardiovascular or noncardiovascular causes.** Type 1 myocardial infarction (red), type 2 myocardial infarction (gold), acute myocardial injury (dark blue), and chronic myocardial injury (light blue). All causes of death which occurred in 5 or more patients are included, with the width of the band indicating the relative size of the population. COPD indicates chronic obstructive pulmonary disease; and MI, myocardial infarction.

Death from any cause occurred in 9% (4367/48 282) of patients, with those with acute myocardial injury at highest risk (Figure II in the online-only Data Supplement). The proportion of deaths from cardiovascular and noncardiovascular causes differed across diagnostic categories (Figure 4). The risk and rate of death from a noncardiovascular cause was highest in patients with acute myocardial injury (Figure 2; csHR 2.65 [95% CI, 2.33–3.01]), type 2 myocardial infarction (csHR 1.72 [95% CI, 1.44–2.06]), and chronic myocardial injury (csHR 2.06 [95% CI, 1.77–2.40]), and was lowest in patients with type 1 myocardial infarction (csHR 0.83 [95% CI, 0.72–0.96]).

### Implementation of the High-Sensitivity Assay

In the implementation phase, the use of high-sensitivity troponin increased the diagnosis of type 1 myocardial infarction by 11% (510/4471), type 2 myocardial infarction by 22% (205/916), acute myocardial injury by 36% (443/1233), and chronic myocardial injury by 43% (389/898). Despite increases in the use of antiplatelet and anticoagulant therapies (43% vs61%) and coronary revascularization (35% vs44%), there was no reduction in the primary outcome in patients with type 1 myocardial infarction (csHR 1.00 [95% CI, 0.82–1.21]; Tables IV and V and Figures III and IV in the online-only data Supplement). Implementation was not associated with additional treatment or improvement in outcomes for patients with type 2 myocardial infarction or myocardial injury (Tables IV and V and Figures III and IV in the online-only data Supplement).

## Discussion

In a randomized, controlled trial, we evaluated the effect of implementing a hs-cTnI assay and the recommendations of the Universal Definition of Myocardial Infarction on clinical outcomes in consecutive patients with suspected acute coronary syndrome. Whilst the majority of patients had a diagnosis of type 1 myocardial infarction, the introduction of high-sensitivity troponin testing led to a disproportionate increase in the diagnosis of type 2 myocardial infarction and myocardial injury. All patients with myocardial injury or infarction were at increased risk of future myocardial infarction or cardiovascular death, with those with type 1 myocardial infarction at highest risk. Despite modest increases in coronary revascularization and preventative therapies in patients with type 1 myocardial infarction, there was no reduction in future cardiovascular events. In patients with type 2 myocardial infarction or myocardial injury, cardiovascular event rates and future risk was similar to patients with type 1 myocardial infarction, despite a marked increase in noncardiovascular death. Here, we observed no change in cardiovascular investigations or treatments, and outcomes were similarly unchanged.

The observed excess in mortality in patients with type 2 myocardial infarction or myocardial injury is consistent with previous observational studies.^8,11,13,19–28^ Patients with acute myocardial injury were at highest risk of noncardiovascular death, with more than a third occurring within 30 days because of pneumonia, an infective exacerbation of chronic obstructive pulmonary disease, or sepsis. In a model attempting to account for this competing risk of noncardiovascular death, patients with type 2 myocardial infarction or myocardial injury had lower rates of cardiovascular events than those with type 1 myocardial infarction. Despite this, 1 in 6 had a myocardial infarction or died from a cardiovascular cause at 1 year; an event rate over 3-fold higher than observed in those without evidence of myocardial injury.

Whilst hs-cTn clearly provides important prognostic information, implementation of testing into practice did not improve outcomes. This may be because in patients with type 2 myocardial infarction or myocardial injury, there is little consensus on how to investigate or treat either group. Even in those with type 1 myocardial infarction, where we have clear guidelines for investigation and treatment, we observed only modest increases in coronary angiography, antiplatelet, or other preventative therapies. This may reflect clinician uncertainty in whether small increases in cardiac troponin are important, and if the benefits of invasive management outweigh the risks in this group. Indeed, our evidence base for the management of myocardial infarction largely predates the introduction of the Universal Definition, when the diagnostic threshold for myocardial infarction was almost 10-fold higher than the threshold implemented in this trial.

The lack of a specific cardiac biomarker to distinguish type 1 myocardial infarction and the increasing frequency of type 2 myocardial infarction and myocardial injury poses challenges to clinicians in practice on a daily basis. As observed in this trial, type 2 myocardial infarction and myocardial injury are responsible for almost half of all elevations in cardiac troponin concentration, and have been shown to be more frequent than type 1 myocardial infarction in hospitalized patients over the age of 75.^13^ Less than half of these patients are referred to cardiology, with the majority managed by general physicians,^13^ and a lack of evidence has led to inconsistency in investigation and treatment.

The classification of type 2 myocardial infarction and myocardial injury is based on expert consensus, and to date no prospective clinical trials have evaluated the utility of this classification.^29^ These conditions arise because of a wide range of pathologies including coronary or structural heart disease, arrhythmias, myocarditis, and many noncardiac conditions.^8^ The latest guidance, requiring evidence of myocardial ischemia and oxygen supply-demand imbalance, has reduced the frequency of type 2 myocardial infarction. Our observations add to previous studies suggesting that any myocardial injury is prognostically important,^11,30,31^ irrespective of whether myocardial ischemia was present, but strategies to guide further investigation and treatment in patients without type 1 myocardial infarction require prospective evaluation.

Whilst implementation of high-sensitivity troponin and the recommendations of the Universal Definition did not improve outcomes here, the proposed framework of classification could be helpful as it encourages clinicians to consider the underlying mechanism of myocardial injury, and to not dismiss troponin elevation as mere bystander injury of no consequence.

The Fourth Universal Definition states patients with type 2 myocardial infarction should not have evidence of acute atherothrombosis, which may encourage clinicians to undertake additional coronary investigations. If recognition of type 2 myocardial infarction or acute myocardial injury during another illness leads to the identification and treatment of previously unrecognized coronary or structural heart disease, it is plausible that cardiovascular outcomes could improve.^16^ Indeed there is increasing evidence that the presence of obstructive coronary disease is the strongest predictor of future adverse cardiovascular outcomes.^11,30,31^ Identification of those with chronic myocardial injury may also be useful, as this may indicate the presence of unrecognized stable cardiovascular disease. A prospective trial of coronary and cardiac imaging in type 2 myocardial infarction is in progress (DEMAND-MI, ClinicalTrials.gov NCT:03338504), and a randomized, controlled trial of coronary investigation and targeted preventative therapy is planned.^32^ These studies are an appropriate first step in the understanding of this heterogeneous group of patients and will help to refine the classification and provide clearer guidance for clinicians in practice. Randomized, controlled trials of secondary prevention therapy are urgently required, but until such data are available to inform clinical guidelines, clinicians should carefully assess cardiovascular risk on an individual patient basis to guide investigation and secondary prevention.

There are several strengths and limitations of our study. This was a prespecified secondary analysis of a randomized, controlled trial, enrolling consecutive patients with suspected acute coronary syndrome across 10 hospitals irrespective of age, sex, time of presentation and severity of illness. As such, we believe our results are generalizable and reflective of real-world clinical practice. Using all available clinical information, we reviewed and classified all patients according to the latest recommendations from the Universal Definition of Myocardial Infarction to ensure our findings are relevant to current practice. Where there was consensus amongst the adjudication panel that there was insufficient clinical information to make a definitive diagnosis, because of missing admission or discharge letters, we did not attempt to adjudicate the diagnosis (1245/10 360, 12%). We had access to all other information including past medical history, clinical investigations, management and outcomes, and provide data for all primary and secondary outcomes in this group within the data supplement (Tables I and II in the online-only Data Supplement). Our trial population was restricted to patients who had cardiac troponin measured for suspected acute coronary syndrome, and we acknowledge that the prevalence of type 2 myocardial infarction and myocardial injury may differ in consecutive patients where cardiac troponin was measured for any reason.^33,34^ Whilst we implemented the recommendations of the Third Universal Definition in our trial, as the Fourth Universal Definition provides no additional guidance on the investigation or treatment of patients with type 2 myocardial infarction or myocardial injury, we think it is unlikely that outcomes would differ had this guideline been in place at the start of the trial. Whilst there was substantial overall agreement between adjudicators for the classification of myocardial injury and infarction, we recognize that agreement was only moderate for the classification of type 2 myocardial infarction or myocardial injury. In addition, we acknowledge that investigations and treatments were undertaken at the discretion of the treating clinician there is a risk of diagnostic misclassification. A formal comparison of the adjudicated diagnosis and ICD-10 coded clinical diagnosis is planned. Serial cardiac troponin measurements were only undertaken in 77% (6983/9115) of patients, which has particular relevance to the distinction between acute and chronic myocardial injury. Consequently, we considered both diagnoses as a single entity when comparing outcomes by study phase to avoid selection bias.

In conclusion, implementation of the recommendations of the Universal Definition of Myocardial Infarction identified patients at high risk of cardiovascular and noncardiovascular events, but was not associated with consistent increases in treatment or improved outcomes. Effective strategies for the investigation and treatment of patients with type 2 myocardial infarction and myocardial injury are required if we are to improve outcomes.

## Acknowledgments

The High-STEACS Investigators contributed to the conception or design of the work, or the acquisition, analysis, or interpretation of data for the work. They were involved in drafting and revising the article and have given final approval of the version to be published. The High-STEACS investigators are accountable for the work. We thank researchers from the Emergency Medicine Research Group Edinburgh for their support during the conduct of this trial.

## Sources of Funding

This trial was funded by a Special Project Grant from the British Heart Foundation (SP/12/10/29922) with additional support from a British Heart Foundation-Turing Cardiovascular Data Science Award (BCDSA/100003) and British Heart Foundation Research Excellence Award (RE/18/5/34216; RE/18/6/3421). Drs Chapman, Mills, and Newby are supported by Clinical Research Training Fellowship (FS/16/75/32533), Butler Senior Clinical Research Fellowship (FS/16/14/32023), and Chair (CH/09/002) awards from the British Heart Foundation. Dr Chapman also receives support from a Starter Grant for Clinical Lecturers from the Academy of Medical Sciences (SGL021\1075). Dr Weir was supported by the National Health Service (NHS) in Lothian through the Edinburgh Clinical Trials Unit. Abbott Laboratories provided cardiac troponin assay reagents, calibrators, and controls without charge. The funders played no role in the design, conduct, data collection, analysis or reporting of the trial.

## Disclosures

Drs Shah and Chapman have received honoraria from Abbott Diagnostics. Dr Mills has received honoraria from Abbott Diagnostics, Siemens Healthineers, and Singulex, and the University of Edinburgh has received research grants from Abbott Diagnostics and Siemens Healthineers. Dr Apple has received honoraria (advisory board) from Siemens Healthineers and LumiraDx; nonsalaried research funding through research foundation from Abbott Diagnostics, Abbott PC, Beckman Coulter, Siemens Healthineers, Quidel, Ortho-Clinical Diagnostics, Roche Diagnostics; Board of Directors HyTest. Dr Berry is named on institutional research or consultancy agreements between the University of Glasgow and Abbot Vascular, AstraZeneca, Coroventis, Corstem, G.S.K., Heartflow, Menarini, Neosoft, Novartis, Philips, and Siemens Healthcare. These companies were not involved in this article. The other authors report no conflicts.

## Supplementary Material
