# Supplementary material for: High-Sensitivity Cardiac Troponin and the Universal Definition of Myocardial Infarction
Source: Circulation. 2019 Oct 7;141(3):161–71. doi: 10.1161/CIRCULATIONAHA.119.042960 (PMC6970546; doi:10.1161/CIRCULATIONAHA.119.042960)
Supplement: Supplementary file 1 [file cir-141-161-s001.pdf]

SUPPLEMENTARY DATA

High-Sensitivity Cardiac Troponin and the Universal Definition of Myocardial Infarction

Andrew R. Chapman, MD.,[1] Philip D. Adamson, MD.,[1,2] Anoop S.V. Shah, MD.,[1] Atul Anand, MD.,[1] Fiona E. Strachan, PhD.,[1] Amy V. Ferry, BSc.,[1] Kuan Ken Lee, MD,[1] Colin Berry, MD,[3] Iain Findlay, MD,[4] Anne Cruikshank, MD,[5] Alan Reid, MSc,[5] Alasdair Gray, MD,[6] Paul O. Collinson, MD,[7] Fred Apple, PhD.,[8] David A. McAllister, MD,[9] Donogh Maguire, MD,[10] Keith A.A. Fox, MD.,[1] Catalina A. Vallejos PhD,[11,12] Catriona Keerie, MSc,[13,14] Christopher J. Weir, PhD,[13,14] David E. Newby, MD.,[1] and Nicholas L. Mills, MD[1,14];
on behalf of the High-STEACS Investigators†

[1] BHF Centre for Cardiovascular Science, University of Edinburgh, Edinburgh, UK.
[2] Christchurch Heart Institute, University of Otago, Christchurch, NZ
[3] Institute of Cardiovascular and Medical Sciences, University of Glasgow, Glasgow, UK.
[4] Department of Cardiology, Royal Alexandra Hospital, Paisley, UK.
[5] Department of Biochemistry, Queen Elizabeth University Hospital, Glasgow, UK.
[6] Emergency Medicine Research Group Edinburgh, Royal Infirmary of Edinburgh, Edinburgh, UK.
[7] Departments of Clinical Blood Sciences and Cardiology, St George's, University Hospitals NHS Trust and St George's University of London, London, UK.
[8] Department of Laboratory Medicine and Pathology, Hennepin Healthcare/Hennepin County Medical Center & University of Minnesota, Minneapolis, MN, USA.
[9] Institute of Health and Wellbeing, University of Glasgow, Glasgow, UK.
[10] Emergency Medicine Department, Glasgow Royal Infirmary, Glasgow, UK.
[11] MRC Human Genetics Unit, University of Edinburgh, Edinburgh UK.
[12] The Alan Turing Institute, London, UK.
[13] Edinburgh Clinical Trials Unit, University of Edinburgh, Edinburgh, UK.
[14] Usher Institute of Population Health Sciences and Informatics, University of Edinburgh, Edinburgh, UK.

†Listed in the Supplementary Appendix

Corresponding Author:
Dr Andrew R Chapman
BHF/University Centre for Cardiovascular Science
The University of Edinburgh
Edinburgh EH16 4SA
United Kingdom
Telephone: 0044 131 242 6515
Fax: 0044 131 242 6379
E-mail: a.r.chapman@ed.ac.uk

Supplementary figures: 4

Supplementary tables: 5

Supplementary appendix: 4

Table S1. Characteristics of All Trial Participants Classified by the Fourth Universal Definition of Myocardial Infarction

	All patients	No myocardial injury	Type 1 myocardial infarction	Type 2 myocardial infarction	Acute myocardial injury	Chronic myocardial injury	Unable to classify
No. of participants	48,282	37,922	4,981	1,121	1,676	1,287	1,245
Age (years), mean (SD)	61 (17)	58 (17)	68 (14)	74 (14)	75 (15)	74 (15)	73 (15)
Men, n (%)	25,720 (53)	20,351 (54)	2,995 (60)	501 (45)	664 (40)	536 (42)	642 (52)
Phase							
Validation	18,978 (39)	14,862 (39)	1,794 (36)	405 (36)	683 (41)	498 (39)	724 (58)
Presenting symptom*							
Chest pain, n (%)	34,540 (81)	28,091 (84)	4,061 (89)	749 (73)	569 (38)	559 (49)	465 (50)
Dyspnoea, n (%)	2,175 (5)	1,107 (3)	171 (4)	116 (11)	372 (25)	235 (21)	173 (19)
Palpitation, n (%)	1,269 (3)	991 (3)	17 (<1)	67 (6)	97 (6)	42 (4)	54 (6)
Syncope, n (%)	2,495 (6)	1,809 (5)	102 (2)	38 (4)	240 (16)	179 (16)	126 (14)
Other, n (%)	2,188 (5)	1,458 (4)	221 (5)	61 (6)	217 (15)	116 (10)	115 (12)
Past medical history							
Myocardial infarction, n (%)	4,214 (9)	2,835 (7)	667 (13)	163 (15)	161 (10)	205 (16)	157 (13)
Ischemic heart disease, n (%)	11,912 (25)	8,455 (22)	1,519 (30)	454 (40)	509 (30)	492 (38)	439 (35)
Cerebrovascular disease, n (%)	2,949 (6)	1,915 (5)	368 (7)	135 (12)	192 (11)	167 (13)	171 (14)
Diabetes mellitus, n (%)	3,518 (7)	2,040 (5)	802 (16)	147 (13)	208 (12)	164 (13)	148 (12)
Heart failure hospitalization, n (%)	4,322 (9)	2,159 (6)	792 (16)	292 (26)	410 (24)	363 (28)	299 (24)
Previous revascularization							
PCI, n (%)	3,682 (8)	2,744 (7)	487 (10)	97 (9)	94 (6)	128 (10)	93 (7)
CABG, n (%)	782 (2)	534 (1)	105 (2)	32 (3)	45 (3)	34 (3)	31 (2)
Medications at presentation							
Aspirin, n (%)	13,163 (27)	9,462 (25)	1,694 (34)	471 (42)	608 (36)	452 (35)	432 (35)
Dual anti-platelet therapy, n (%)†	1,605 (3)	1,103 (3)	233 (5)	64 (6)	71 (4)	68 (5)	55 (4)
Statin, n (%)	19,366 (40)	14,106 (37)	2,377 (48)	632 (56)	852 (51)	686 (53)	669 (54)
ACE inhibitor or ARB, n (%)	15,618 (32)	11,285 (30)	1,995 (40)	514 (46)	692 (41)	579 (45)	523 (42)
Beta-blocker, n (%)	13,173 (27)	9,566 (25)	1,598 (32)	489 (44)	564 (34)	460 (36)	465 (37)
Oral anti-coagulant, n (%)‡	3,253 (7)	2,158 (6)	292 (6)	170 (15)	225 (13)	198 (15)	208 (17)
Electrocardiogram§							
Normal	-	-	1,578 (32)	201 (18)	400 (24)	363 (28)	107 (9)
Myocardial ischemia	-	-	1,872 (38)	383 (34)	112 (7)	75 (6)	49 (4)
ST-segment elevation	-	-	870 (17)	36 (3)	38 (2)	40 (3)	5 (<1)
ST-segment depression	-	-	865 (17)	278 (25)	87 (5)	56 4)	36 (3)
T-wave inversion	-	-	780 (16)	166 (15)	128 (8)	148 (11)	46 (4)
Physiological parameters§							
Heart rate, beats per minute	-	-	79 (20)	105 (35)	94 (29)	85 (24)	90 (27)

Systolic blood pressure, mmHg	-	-	142 (28)	132 (30)	136 (31)	137 (29)	134 (30)
Hematology and clinical chemistry							
Hemoglobin, g/L	136 (22)	137 (20)	136 (22)	126 (29)	128 (25)	127 (24)	126 (28)
eGFR, ml/min	54 (13)	56 (10)	51 (14)	46 (15)	45 (16)	45 (17)	45 (18)
Peak hs-cTnI, ng/L	4 [2, 16]	3 [1, 6]	855 [104, 6775]	125 [48, 604]	74 [37, 307]	55 [34, 145]	38 [25, 136]

Presented as mean (SD), median (inter-quartile range), or number (%). There were significant differences (P<0.001) between groups for all co-variates. P values obtained from group-wise comparisons using Chi-square, Kruskal Wallis or one way analysis of variance tests as appropriate. Cell counts <5 are redacted in line with regulatory approvals.

*Presenting symptom was missing in 5,615 (12%) patients.

†Two medications from aspirin, clopidogrel, prasugrel or ticagrelor. ‡Includes warfarin or novel oral anti-coagulants.

§ Electrocardiographic findings and physiological parameters only reported for those with elevation in cardiac troponin concentrations.

Abbreviations: ACE = angiotensin converting enzyme; ARB = angiotensin receptor blockers; eGFR = estimated glomerular filtration rate; CABG = coronary artery bypass grafting; PCI = percutaneous coronary intervention.

Table S2. Primary and Secondary Outcomes at 1 year Stratified by the Fourth Universal Definition of Myocardial Infarction

	All patients	No myocardial injury	Type 1 myocardial infarction	Type 2 myocardial infarction	Acute myocardial injury	Chronic myocardial injury	Unable to classify
No. of participants	48,282	37,922	4,981	1,121	1,676	1,287	1,245
Primary outcome							
Myocardial infarction* or cardiovascular death	2,586 (5)	846 (2)	863 (17)	162 (14)	273 (16)	207 (16)	226 (18)
Secondary outcomes							
Myocardial infarction*	1,046 (2)	361 (1)	466 (9)	51 (5)	56 (3)	57 (4)	48 (4)
All-cause death	4,367 (9)	1,994 (5)	720 (14)	258 (23)	561 (33)	374 (29)	456 (37)
Cardiovascular death	1,693 (4)	516 (1)	479 (10)	120 (11)	230 (14)	164 (13)	182 (15)
Cardiac death	1,273 (3)	334 (1)	431 (9)	100 (9)	157 (9)	129 (10)	120 (10)
Non-cardiovascular death	2,673 (6)	1,478 (4)	241 (5)	138 (12)	331 (20)	209 (16)	274 (22)
Heart failure hospitalization	1,700 (4)	671 (2)	380 (8)	134 (12)	180 (11)	183 (14)	148 (12)
Ischemic stroke	546 (1)	344 (1)	75 (2)	18 (2)	38 (2)	37 (3)	33 (3)
Safety end points							
Major haemorrhage†	195 (<1)	95 (<1)	39 (1)	14 (1)	16 (1)	9 (1)	22 (2)
Unplanned hospitalization§	8,489 (18)	5,445 (14)	1,616 (32)	218 (19)	360 (21)	236 (18)	605 (49)

Presented as number (SD). * Type 1 or type 4b myocardial infarction. † Bleeding Academic Research Consortium (BARC) type 3 or type 5
§ Unplanned hospitalization excluding for acute coronary syndrome at 30 days. Cell counts <5 are redacted in line with regulatory approvals.

Table S3. Cause-specific hazard ratios for the primary outcome and non-cardiovascular death for patients with myocardial injury and infarction versus patients with no myocardial injury

	Myocardial infarction or cardiovascular death		Non-cardiovascular death	
	**Unadjusted csHR (95% CI)**	**Adjusted csHR (95% CI)**	**Unadjusted csHR (95% CI)**	**Adjusted csHR (95% CI)**
Type 1 myocardial infarction	8.62 (7.84 to 9.48)	5.64 (5.12 to 6.22)	1.24 (1.08 to 1.43)	0.83 (0.72 to 0.96)
Type 2 myocardial infarction	7.24 (6.12 to 8.56)	3.50 (2.94 to 4.15)	3.54 (2.97 to 4.23)	1.72 (1.44 to 2.06)
Acute myocardial injury	8.91 (7.77 to 10.21)	4.38 (3.80 to 5.05)	6.27 (5.56 to 7.07)	2.65 (2.33 to 3.00)
Chronic myocardial injury	8.33 (7.16 to 9.70)	3.88 (3.31 to 4.55)	4.88 (4.22 to 5.65)	2.06 (1.77 to 2.40)

Cause-specific hazard ratios (csHR) obtained from unadjusted and multivariable cox regression models including adjustment for age, sex, a history of diabetes or ischemic heart disease, season, days since the start of the trial, creatinine value at presentation and site of recruitment (as a random effect). In this model the competing event or time of censor are both considered as independent outcomes.

Table S4. Management During Index Hospital Admission Stratified by classification and study phase

	Type 1 myocardial infarction		Type 2 myocardial infarction		Acute and chronic myocardial injury	
	**Validation**	**Implementation**	**Validation**	**Implementation**	**Validation**	**Implementation**
No. of participants	1,794	3,187	405	716	1,181	1,782
Duration of stay, hrs	75 [26, 134]	74 [45, 124]	99 [44, 212]	94 [34, 195]	131 [27, 311]	102 [29, 262]
ACS treatment in ED	771 (43)	1,946 (61)	98 (24)	196 (27)	123 (10)	285 (16)
New anti-platelet therapy	1,151 (64)	2,203 (69)	74 (18)	135 (19)	142 (12)	208 (12)
New dual anti-platelet therapy‡	991 (55)	1,978 (62)	43 (11)	73 (10)	74 (6)	96 (5)
Coronary angiography†	969 (54)	1,959 (61)	38 (9)	77 (11)	42 (4)	93 (5)
PCI	620 (35)	1,401 (44)	5 (1)	12 (2)	<5	<5
New ACE inhibitor or ARB therapy	560 (31)	1,017 (32)	37 (9)	67 (9)	68 (6)	120 (7)
New beta-blocker therapy	643 (36)	1,235 (39)	59 (15)	160 (22)	116 (10)	212 (12)
New statin therapy	573 (32)	1,191 (37)	25 (6)	43 (6)	58 (5)	80 (4)
New oral anticoagulant	46 (3)	83 (3)	64 (16)	145 (20)	88 (7)	143 (8)

Values are number (%) or median (inter-quartile range).
Abbreviations: ACE = angiotensin converting enzyme; ARB = angiotensin receptor blockers; CABG = coronary artery bypass grafting; ED = Emergency Department; PCI = percutaneous coronary intervention.
Cell counts <5 are redacted in line with regulatory approvals.
† Angiography and revascularization within 30 days of presentation
‡ Two medications from aspirin, clopidogrel, prasugrel or ticagrelor

Table S5. Cause-specific hazard ratio for the primary outcome by implementation versus validation phase

	All patients	
	**Implementation versus validation phase ***	**P-value for interaction**
Type 1 myocardial infarction	1.00 (0.82 to 1.21)	0.96
Type 2 myocardial infarction	0.96 (0.68 to 1.36)	0.84
Acute or chronic myocardial injury	0.87 (0.69 to 1.09)	0.23

[*]Adjusted cause-specific hazard ratios (csHR) obtained from multivariable cox regression models including adjustment for age, sex, a history of ischemic heart disease or diabetes mellitus, renal function, time of presentation from the start date of the trial, season, site of recruitment (as a random effect), log transformed peak troponin concentration, phase of the trial, diagnostic classification, and an interaction term for phase of the trial and diagnostic classification. In this model the competing event or time of censor are both considered as independent outcomes.

Figure S1. Classification by Third and Fourth Universal Definition of Myocardial Infarction

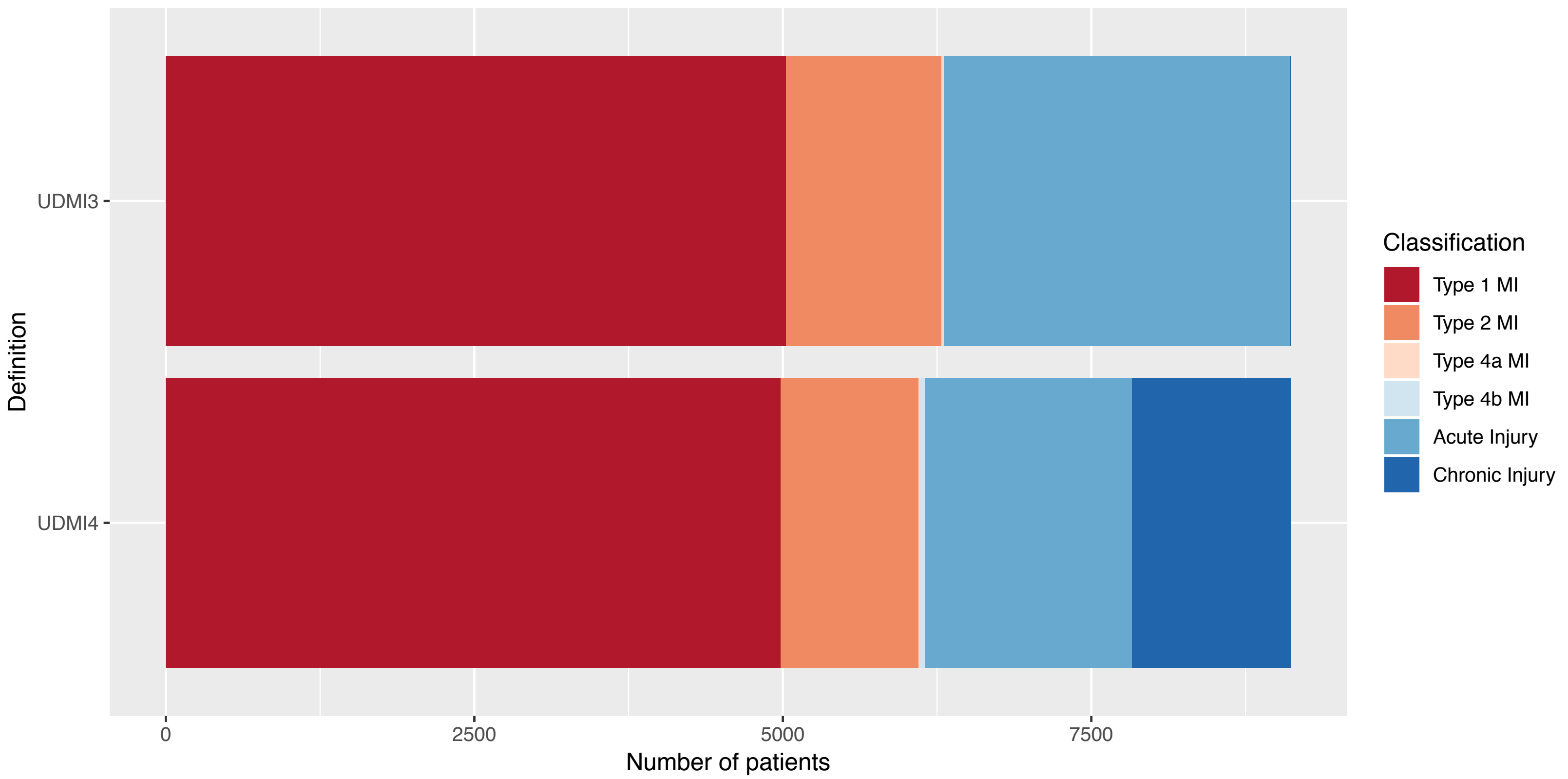

	Type 1 MI	Type 2 MI	Type 4a MI	Type 4b MI	Acute Injury	Chronic Injury
**UDMI3**	5028	1260	9	8	2810	NA
**UDMI4**	4981	1121	9	41	1676	1287

Figure S2. Cumulative incidence of primary outcome and all-cause death with table of number at risk.

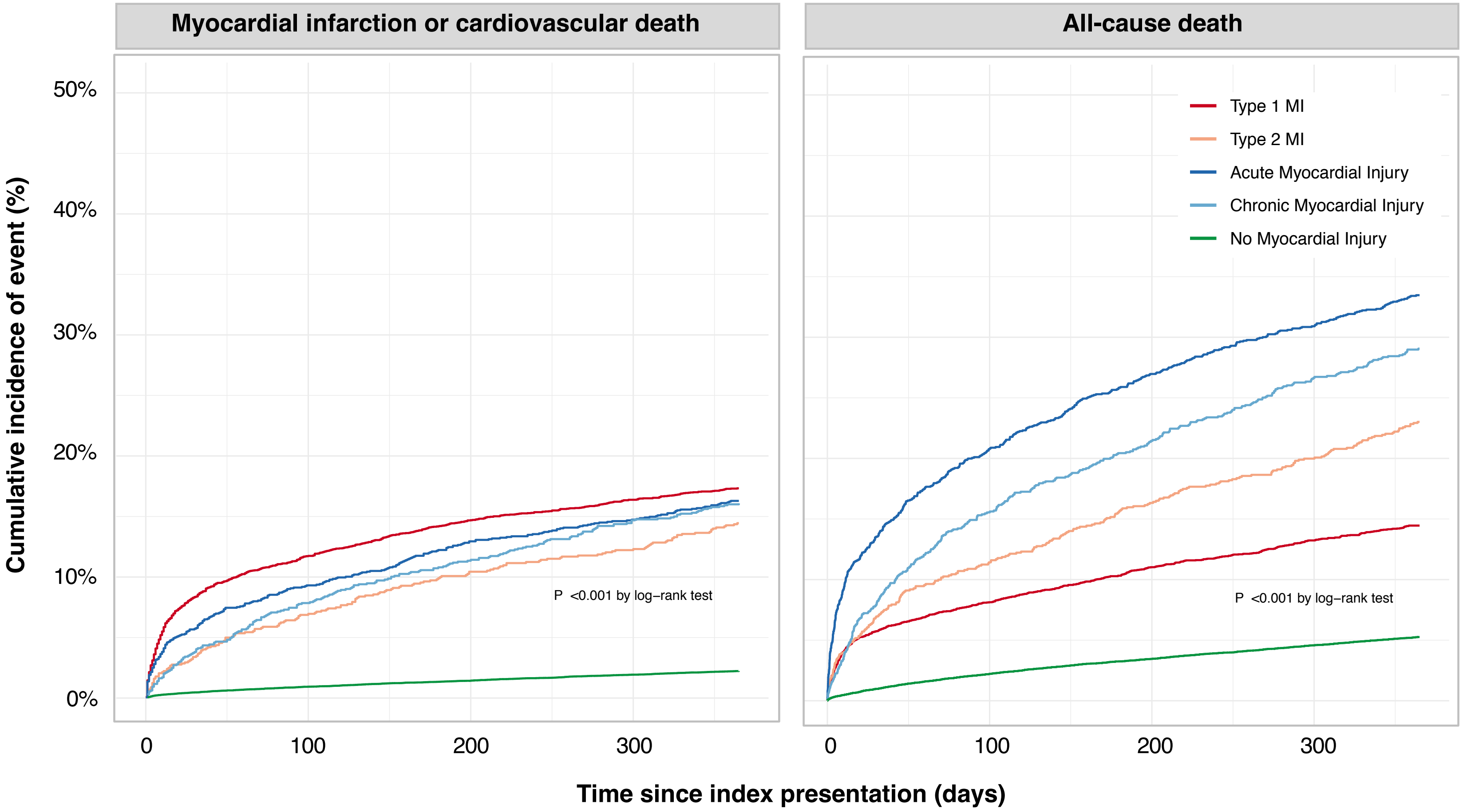

**Type 1 MI**	4981	4397	4250	4165	4981	4576	4432	4322
**Type 2 MI**	1121	1044	1004	984	1121	993	938	897
**Acute Myocardial Injury**	1676	1520	1459	1429	1676	1328	1224	1158
**Chronic Myocardial Injury**	1287	1186	1141	1098	1287	1087	1011	944
**No Myocardial Injury**	37922	37569	37381	37190	37922	37084	36613	36186

Figure S3. Forest plot of the primary outcome by study phase and classification.

		Validation phase		Implementation phase	
		n	%	n	%
Myocardial infarction or cardiovascular death	Type 1 MI	332	18.5	531	16.7
	Type 2 MI	64	15.8	98	13.7
	Myocardial Injury	215	18.2	265	14.9

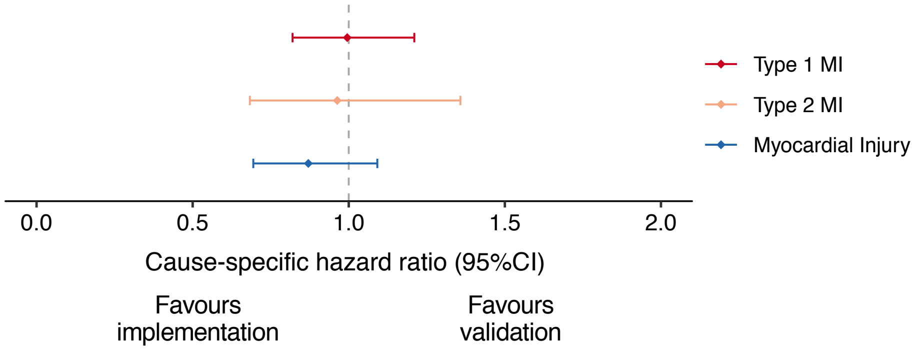

Figure S4. Kaplan-Meier curves of the primary outcome by classification

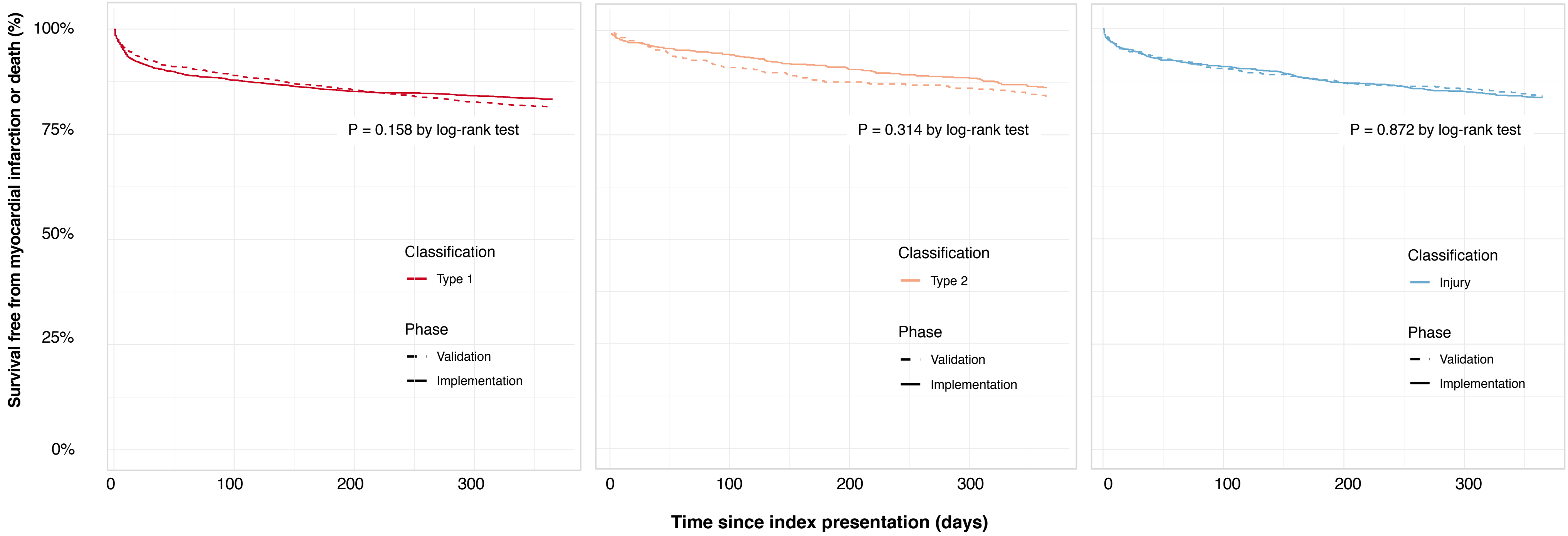

**Validation**	1794	1596	1535	1483	405	369	355	349	683	617	594	585
**Implementation**	3187	2801	2715	2682	716	675	649	635	993	903	865	844

Figure legends

Figure S1. Distribution of patients classified by the Third and Fourth Universal Definition of Myocardial Infarction. Number of patients classified by third universal definition: type 1 myocardial infarction = 5,028, type 2 myocardial infarction = 1,260, type 4a myocardial infarction = 9, type 4b myocardial infarction = 8, acute myocardial injury = 2,810, chronic myocardial injury = 0. Number of patients classified by fourth universal definition: type 1 myocardial infarction = 4,981, type 2 myocardial infarction = 1,121, type 4a myocardial infarction = 9, type 4b myocardial infarction = 41, acute myocardial injury = 1,676, chronic myocardial injury = 1,287. A total of 885 patients could not be classified as the adjudicators reported there was insufficient clinical information.

Figure S2. Cumulative incidence curves for the primary outcome of type 1 or 4b myocardial infarction or cardiovascular death, and all-cause death, stratified by type 1 myocardial infarction (red), type 2 myocardial infarction (gold), acute myocardial injury (dark blue), chronic myocardial injury (light blue) and no myocardial injury (green) with table of number at risk.

Figure S3. Forest plot of the primary outcome (type 1 or 4b myocardial infarction or cardiovascular death) in the trial population by study phase, stratified by index diagnosis; type 1 myocardial infarction (red), type 2 myocardial infarction (gold) and acute or chronic myocardial injury (blue). As serial sampling was not available in all patients in both phases of the trial, acute and chronic myocardial injury are considered as a single diagnosis. Adjusted cause-specific hazard ratios (csHR) obtained from multivariable cox regression models including adjustment for age, sex, a history of diabetes or ischemic heart disease, season, days since the start of the trial, creatinine value at presentation, site of recruitment (as a random effect), trial phase, diagnostic classification, and an interaction term for trial phase and diagnostic classification.

Figure S4. Kaplan-meier curves for the primary outcome of type 1 or 4b myocardial infarction or cardiovascular death by classification of myocardial infarction with table of number at risk. These estimates do not account for competing risk.

Supplementary Appendix A. Summary of procedures in the High-STEACS trial.

Randomization

Block randomisation was used with sites paired based on the expected number of presentations and one site randomised to early implementation and the other to late implementation. For pragmatic reasons (shared lab facilities out of hours), the Vale of Leven and Royal Alexandra Hospital, Paisley were grouped and randomised together. This enabled implementation of the high-sensitivity assay to occur on the same date at both sites and allowed the same lab processes to be followed at both sites. The randomisation sequence was generated by a programmer at the Edinburgh Clinical Trials Unit who was not otherwise involved in the study using computer generated pseudo-random numbers.

Intervention

Cardiac troponin testing was performed at presentation and was repeated 6 or 12 h after the onset of symptoms at the discretion of the attending physician and in accordance with national guidelines (14). In the validation phase and implementation phase, a contemporary cardiac troponin I (cTnI) assay (Abbott Laboratories, Abbott Park, IL, USA) and a hs-cTnI assay (ARCHITECT$_{STAT}$ high-sensitive troponin I assay; Abbott Laboratories, Abbott Park, IL, USA), respectively, were used to guide clinical decisions. The inter-assay coefficient of variation was determined at each site and was less than 10% at 40 ng/L (seven sites) and 50 ng/L (three sites). Only cTnI concentrations above these diagnostic thresholds were reported. During the implementation phase, a hs-cTnI assay (ARCHITECT$_{STAT}$ high-sensitive troponin I assay; Abbott Laboratories, Abbott Park, IL, USA) was used to guide clinical decisions. This assay has an inter-assay coefficient of variation of less than 10% at 4.7 ng/L, and a 99th centile upper reference limit of 34 ng/L in men and 16 ng/L in women (1).

Throughout the trial period contemporary and high-sensitivity troponin assays were run simultaneously in plasma surplus to clinical requirements, with clinicians blinded to the results of the high-sensitivity assay during the validation phase, and the contemporary assay during the implementation phase. All sites reported cardiac troponin using a contemporary troponin assay and existing diagnostic threshold in a validation phase of at least 6 months, before being randomly allocated to early or late implementation of the high-sensitivity assay with sex-specific thresholds for the diagnosis of myocardial infarction.

Implementation support

To support implementation, we provided written educational material and presentations at each site, training for clinical and laboratory staff, and we updated the electronic patient record to highlight the change in assay and diagnostic thresholds. Educational material on the new assay, decision thresholds and diagnosis of myocardial infarction was presented at each Emergency Department handover (twice daily) during the implementation phase to ensure wide coverage of staff on all shift patterns. This was reinforced by specialist chest pain nurses who received detailed training prior to implementation and who support Emergency Department clinicians in the assessment of patients with suspected acute coronary syndrome.

Key details from the educational presentation formed a one-page reference guide that was posted within each department and online in the hospital guidelines portal. This included guidance for diagnosis as per the universal definition of myocardial infarction. We encouraged clinicians to select only patients with suspected acute coronary syndrome for testing, and to consider aetiology, including a detailed summary of the range of cardiac and non-cardiac conditions which may be responsible for myocardial injury. This information was also

presented to the wider hospital teams in medical grand round presentations prior to implementation, and circulated to all general practitioners.

Every high-sensitivity cardiac troponin result reported in the electronic health record during the implementation phase was accompanied with guidance notes outlining the new assay, reporting units and thresholds. Laboratory staff also received training to ensure any queries directed to the laboratory were dealt with consistently. Finally, the research team included senior cardiologists, emergency physicians, and cardiology nurses who are clinically active within each of the hospital clusters; education was therefore reinforced at a local level by these clinical leaders throughout the implementation phase.

Outcomes

All in-hospital and community deaths, and all hospital admissions are recorded on the Register of Deaths in Scotland and the Scottish Morbidity Record (SMR) respectively. It is a statutory requirement that any deaths occurring in Scotland, or outwith Scotland but within the United Kingdom are entered on the Register of Deaths in Scotland within eight days of death. As such, this registry is 100% complete for the study population, which was restricted to those resident in Scotland. This makes an assumption that patients did not emigrate in the year following enrolment. However, the Scottish population is very stable, with low levels of emigration outwith the United Kingdom.

The TrakCare software application (InterSystems Corporation, Cambridge, MA, USA) is an electronic patient record system used at all participating sites, which provided clinical data for all subsequent hospital admissions. All attendances across any participating hospital where cardiac troponin was measured and the hs-cTnI concentration was >99th centile were reviewed and the diagnosis adjudicated. We used the same approach to adjudication as for the index

hospital episode with the panel blinded to all cardiac troponin measurements during the index episode and to the study phase.

The primary outcome was myocardial infarction (type 1 or type 4b) or cardiovascular death at 1 year. Secondary efficacy end-points include myocardial infarction, unplanned coronary revascularisation, cardiovascular death, cardiac death, all-cause death, duration of stay, hospitalisation for heart failure, and ischaemic stroke. Secondary safety end-points include major haemorrhage, unplanned hospitalisation excluding acute coronary syndrome, and non-cardiovascular death. The duration of stay was derived from a common electronic patient record system used across all participating sites (TrakCare, InterSystems Corporation, Cambridge, MA, USA) and was calculated from the admission and discharge date and time to the nearest minute.

Unplanned coronary revascularisation was defined as any urgent or emergency percutaneous coronary intervention or coronary artery bypass grafting following discharge. International Classification of Disease (ICD)-10 codes from the Scottish Morbidity Record were used to define hospitalisation for heart failure (I50) and ischaemic stroke (I63, I65, or I66). Bleeding was defined according to the Bleeding Academic Research Consortium (BARC) definition using ICD-10 and OPCS codes to classify each bleeding event. Major haemorrhage was defined as BARC type 3 or type 5. Unplanned hospitalisation excluding acute coronary syndrome was defined as any hospital attendance or admission excluding type 1 or type 4b myocardial infarction at 30 days.

The decision to perform coronary angiography was made by the attending cardiologist taking into consideration all aspects of the patients presentation including cardiac troponin concentrations.

The Scottish national community drug-prescribing database of ISD in NHS Scotland maintains a detailed record of all prescriptions dispensed in the community, which are linked to individual patient identifiers. Alterations in cardiovascular therapies following the index hospital episode were determined by comparison to baseline.

Data Sharing

The High-STEACS trial makes use of multiple routine electronic health care data sources that are linked, deidentified and held in our national safe haven, which is accessible by approved individuals who have undertaken the necessary governance training. Summary data can be made available upon request to Nicholas Mills (nick.mills@ed.ac.uk).

Supplementary Appendix B. Detailed description of diagnostic adjudication.

All patients with hs-cTnI concentrations above the sex-specific 99th centile were classified according to the Third Universal Definition of Myocardial Infarction in use at the time of the trial. In this pre-specified secondary analysis, we updated this classification in accordance with the Fourth Universal Definition of Myocardial Infarction. The final diagnosis was adjudicated according to a pre-specified list (cardiac diagnoses: acute aortic dissection, acute heart failure, cardiomyopathy, chronic heart failure, hypertensive heart disease, myopericarditis, non-ST-segment elevation myocardial infarction, ST-segment elevation myocardial infarction, recent myocardial infarction, tachyarrhythmia, takotsubo cardiomyopathy or valvular heart disease; non-cardiac diagnoses: acute kidney injury, chronic kidney disease, chronic obstructive pulmonary disease, gastrointestinal bleed, pulmonary embolism, sepsis, or other).

Two physicians independently reviewed all clinical information, blinded to study phase, with discordant diagnoses resolved by a third reviewer. Clinical information included the dates and times of presentation and final discharge, the initial emergency department assessment and final discharge letter as documented in the electronic care record, with summaries of all investigations undertaken during the index presentation including the electrocardiogram. The adjudication panel had access to raw clinical information including haemoglobin, creatinine and high-sensitivity cardiac troponin I concentrations, and the reports from invasive coronary angiography. Type 1 myocardial infarction was defined as myocardial necrosis (any hs-cTnI concentration above the 99th centile with a rise and/or fall in hs-cTnI concentration where serial testing was performed) in the context of a presentation with suspected acute coronary syndrome with symptoms or signs of myocardial ischemia on the electrocardiogram. Patients with symptoms or signs of myocardial ischemia and evidence of increased oxygen demand or

decreased supply (for example, tachyarrhythmia, hypotension, or anemia) secondary to an alternative pathology and myocardial necrosis were defined as type 2 myocardial infarction. The classification of type 2 myocardial infarction also includes patients with coronary vasospasm, embolism or spontaneous dissection without evidence of atherothrombosis related to coronary artery disease. Type 4a myocardial infarction was defined in patients with symptoms or signs of myocardial ischemia following percutaneous coronary intervention where hs-cTnI concentrations were 5-fold greater than the 99th centile, or increased further if elevated prior to the procedure. Type 4b myocardial infarction was defined where myocardial ischemia and myocardial necrosis were associated with stent thrombosis documented at angiography. Myocardial injury was defined if hs-cTnI concentrations were above the 99th centile in the absence of any clinical features of myocardial ischemia. All non-ischemic myocardial injury was classified as acute, unless a change of <20% was observed on serial testing or the final adjudicated diagnosis was chronic heart failure or chronic renal failure, where the classification was chronic myocardial injury.

Supplementary Appendix C. Pre-specified analysis plan

STATISTICAL ANALYSIS PLAN

High-Sensitivity Troponin in the Evaluation of patients with Acute Coronary Syndrome (High-STEACS): a randomised controlled trial

Secondary Analysis: High-sensitivity cardiac troponin I and the classification of myocardial infarction

Version: 1.1

Date: 26th May 2018

Chief Investigator: Professor Nicholas L Mills

Protocol Author: Dr Andrew R Chapman

Signatures

Chief Investigator
Prof Nicholas L Mills ______________________ Date: 26th May 2018
BHF Centre for Cardiovascular Sciences
University of Edinburgh

Protocol Author
Dr Andrew R Chapman ______________________ Date: 26th May 2018
BHF Centre for Cardiovascular Sciences
University of Edinburgh

Document Version Summary

Version number	Reason for update	Updated by	Date
DRAFT 1.0	Creation of Draft SAP	Andrew Chapman	22 May 2018
1.1	Version for signature	Andrew Chapman	26 May 2018

Table of Contents

Table of Contents **22**

1 Introduction **24**

1.1 Background *24*

1.2 Aims *25*

1.3 Hypothesis *25*

2 Analysis plan **25**

2.1 Design *25*

2.2 Participants *26*

2.2.1 Inclusion criteria 26

2.2.2 Study population 26

2.3 Primary outcome *26*

2.4 Secondary outcomes *26*

3 Methods **26**

3.1 Classification *27*

3.2 Outcomes *27*

3.3 Models *27*

3.4 Sensitivity analyses *27*

4 Expected outcomes **28**

Introduction

Background

The development of high-sensitivity cardiac troponin assays has led to improvements in the precision of cardiac troponin measurements at low concentrations, and more accurate detection of the normal reference range in healthy individuals.[1] However, more sensitive troponin assays have also resulted in increasing recognition of myocardial necrosis in conditions other than acute coronary syndrome.[2]

The universal definition of myocardial infarction recommends a classification based on aetiology, where type 1 myocardial infarction is due to plaque rupture or erosion with atherothrombotic consequences, and type 2 myocardial infarction due to myocardial oxygen supply-demand imbalance in the absence of atherothrombosis. Patients with elevated cardiac troponin concentrations who do not have overt myocardial ischemia are classified as having myocardial injury.[3] Whilst these diagnostic categories are considered distinct in guidelines, implementation in clinical practice has been challenging due to similarities between patients with type 2 myocardial infarction and myocardial injury. Both diagnoses are common, and outcomes are extremely poor, with less than one third of patients with type 2 myocardial infarction alive at five years.[4]

In this secondary analysis of the High-STEACS trial, we wish to determine the effect of implementing a high-sensitivity cardiac troponin assay and sex-specific thresholds on the frequency of diagnosis of different subtypes of myocardial infarction when classified in accordance with the universal definition, and whether implementation lead to changes in investigation, management and clinical outcomes for patients with type 2 myocardial infarction or myocardial injury.

Aims

1. To determine the frequency, characteristics and clinical features of patients with myocardial infarction when classified by the universal definition of myocardial infarction.
2. To assess changes in investigations, treatments and clinical outcomes for patients with myocardial injury and infarction subtypes before and after introduction of a high-sensitivity cardiac troponin assay
3. To determine whether the distinction between type 2 myocardial infarction and myocardial injury identifies patients at increased cardiovascular risk
4. To explore outcomes in those with type 2 myocardial infarction and myocardial injury with and without obstructive coronary artery disease (>50% stenosis in an epicardial vessel)

Hypothesis

1. The introduction of high-sensitivity cardiac troponin assays will result in an increase in referral for to cardiology, more invasive coronary angiography and an increase in prescriptions for antiplatelet or anticoagulant drugs for patients with type 2 myocardial infarction or myocardial injury.
2. Changes in investigation and management will lead to improvement in outcomes for patients with type 2 myocardial infarction and myocardial injury, with a reduction in future cardiovascular risk.
3. The classification and distinction between type 2 myocardial infarction and myocardial injury (based on the presence of myocardial ischaemia) will not distinguish future risk of cardiovascular events.
4. The presence of coronary artery disease will be an independent predictor of future cardiovascular events in this group.

Analysis plan

Design

Sub-study of a stepped wedge cluster randomized controlled trial

Participants

Inclusion criteria

All participants enrolled in the HighSTEACS clinical trial with cardiac troponin concentrations >99th centile, classified as per the universal definition of myocardial infarction. A consort diagram will illustrate identification of the study population.

Study population

The HighSTEACS trial has recruited approximately 47,000 patients. Assuming a prevalence of myocardial necrosis of 18%, approximately 8,500 patients will be suitable for inclusion, and an estimated 20% (1,700) patients will be classified with type 2 myocardial infarction.

Primary outcome

- Myocardial infarction (type 1 or type 4b) or cardiovascular death at 1 year.

Secondary outcomes

- Type 1 or type 4b myocardial infarction following the index presentation
- Unplanned coronary revascularization after discharge
- Cardiovascular death
    - Any cardiovascular death
    - Cardiovascular death – Cardiac subset
    - Cardiovascular death – Non-cardiac subset
- All-cause death
- Hospitalisation for heart failure
- Ischaemic stroke
- Major haemorrhage (BARC Type 3 and BARC Type 5)
- Unplanned hospitalization within 30 days excluding acute coronary syndrome
- Non-cardiovascular death

Additional outcomes

- Rehospitalisation with type 2 myocardial infarction or myocardial injury

Methods

Classification

Patients will be grouped based on the adjudicated diagnosis (type 1 – 5 myocardial infarction or myocardial injury). Baseline characteristics, investigations and treatments received will be summarized as mean (SD) or median (IQR) as appropriate based on distribution. The adjudicated mechanism of type 2 myocardial infarction or myocardial injury will be tabulated. The prevalence of myocardial infarction subtype will be reported (n, %) in all patients, and in those reclassified by the high-sensitivity assay.

Outcomes

Event rates will be calculated for primary and secondary outcomes at 1 year, and reported stratified by the index classification for the total population, and in those reclassified by the high-sensitivity assay. Cumulative incidence or Kaplan-meier curves will be constructed for primary and secondary outcomes, stratified by diagnosis with table of number at risk and comparison in event rates between phases using the log-rank test.

Models

Cause-specific hazard ratios will be determined for primary and secondary outcomes using cox regression models to explore competing risks. Models will be adjusted for clinically relevant co-variates including diabetes, hypertension, stroke, known coronary heart disease (previous myocardial infarction or revascularization). In addition, models will adjust for time since the start of the trial, season, phase of the trial and will include hospital site as a random effect. A further cox regression model will evaluate whether implementation of the high-sensitivity assay improved outcomes in patients when classified by the universal definition. All analysis will be performed using the statistical program R (version 3.2.2).

Sensitivity analyses

We will determine whether clinical outcomes differ in patients with known, likely or unlikely coronary artery disease.

Expected outcomes

Determining frequency of investigation and treatments received will inform clinicians of current practice. Evidence of survival benefit for those who receive prescriptions for antiplatelet, anticoagulant or statin therapy may signal need for the design of randomized controlled trials in this population.

References

1. White HD, Thygesen K, Alpert JS, Jaffe AS. Clinical implications of the Third Universal Definition of Myocardial Infarction. *Heart*. 2014;100:424-434.
2. Alpert JS, Thygesen KA. The Case for a Revised Definition of Myocardial Infarction – The Ongoing Conundrum of Type 2 Myocardial Infarction vs Myocardial Injury. *JAMA Cardiol*. 2016;1:249-250.
3. Thygesen K, Alpert JS, Jaffe AS, Simoons ML, Chaitman BR, White HD. Third universal definition of myocardial infarction. *Eur Heart J*. 2012;33:2551-2567.
4. Chapman AR, Shah AS, Lee KK, Anand A et al. Long term outcomes in patients with type 2 myocardial infarction and myocardial injury. *Circulation.* 2018;137;1236-1245.

Supplementary Appendix D

The High-STEACS Investigators

Chief Investigator: Prof Nicholas L Mills.

Trial managers: Dr Fiona E Strachan and Mr Christopher Tuck.

Trial research team: Dr Anoop S V Shah, Dr Fiona E Strachan, Dr Atul Anand, Dr Anda Bularga, Dr Ryan Wereski, Ms Amy V Ferry, Dr Kuan Ken Lee, Dr Andrew R Chapman, Mr Dennis Sandeman, Dr Philip D Adamson, Dr Catherine L Stables, Dr Catalina A Vallejo, Dr Athanasios Tsanasis, Ms Lucy Marshall, Ms Stacey D Stewart, Dr Takeshi Fujisawa, Ms Mischa Hautvast, Ms Jean McPherson and Ms Lynn McKinlay.

Grant applicants: Prof Nicholas L Mills (Principal Applicant), Prof David E Newby, Prof Keith AA Fox, Prof Colin Berry, Dr Simon Walker, and Dr Christopher J Weir.

Trial steering committee: Prof Ian Ford (chair, independent), Prof Nicholas L Mills, Prof David Newby, Prof Alasdair Gray, Prof Keith AA Fox, Prof Colin Berry, Dr Simon Walker, Prof Paul O Collinson, Prof Fred S Apple, Mr Alan Reid, Dr Anne Cruikshank, Dr Iain Findlay, Dr Shannon Amoils (independent), Dr David A McAllister, Dr Donogh Maguire, Ms Jennifer Stevens (independent), Prof John Norrie (independent), and Prof Christopher Weir.

Adjudication panel: Dr Anoop S V Shah, Dr Atul Anand, Dr Andrew R Chapman, Dr Kuan Ken Lee, Dr Jack Andrews, Dr Phil Adamson, Dr Alastair Moss, Dr Mohamed Anwar, Dr John Hung, Prof Nicholas L Mills.

Biochemistry sub-group committee: Dr Simon Walker, Dr Jonathan Malo, Mr Alan Reid, Dr Anne Cruikshank, Prof Paul O Collinson

Data monitoring committee: Prof Colin Fischbacher, Dr Bernard Croal, Prof Stephen J Leslie.

Edinburgh Clinical Trials Unit: Ms Catriona Keerie, Prof Christopher Weir, Mr Richard Parker, Mr Allan Walker, Mr Ronnie Harkess, Mr Chris Tuck, Mr Tony Wackett

NHS Greater Glasgow & Clyde Safe Haven: Dr Roma Armstrong, Ms Marion Flood, Ms Laura Stirling, Ms Claire MacDonald, Mr Imran Sadat, Mr Frank Finlay.

NHS Lothian eHealth and Safe Haven: Dr Heather Charles, Ms Pamela Linksted, Mr Stephen Young, Mr Bill Alexander, Mr Chris Duncan
